# P03 Utilizing a videography platform for the therapeutic management of chronic pain relating to Hypermobility in children and young people

**DOI:** 10.1093/rap/rkac067.003

**Published:** 2022-09-28

**Authors:** Verna Cuthbert, Vicki Leeson, Jen Nisbet

**Affiliations:** Royal Manchester children's Hospital, Manchester, United Kingdom; Royal Manchester children's Hospital, Manchester, United Kingdom; Royal Manchester Children's Hospital, Manchester, United Kingdom

## Abstract

**Introduction/Background:**

Many patients present to Paediatric Rheumatology clinic, (53%) with chronic pain related to Hypermobility. Pre-Covid, an evidenced based hypermobility class was developed and run by the Allied Health Professional team for children and young people (C & YP), and their parents/carers. These 6 classes were group-based and included education and exercise therapy but were stopped due to the pandemic. Feedback gained was good from the class, although reported issues included reduced school attendance, accessibility due to location, parental time off work, financial and time implications of travel, and unsuitability of group interventions for CYP with social anxiety and autism disorders.

**Description/Method:**

A digital platform (Vimeo) was used to present Hypermobility education and exercise content created by the Paediatric Rheumatology team. The video aims to address these barriers, allowing more equality of access for all CYP; digital access allows CYP and parents to re-visit this information at any time, providing ongoing support for long term management of this condition. The digital platform is being developed to maximize the effectiveness of therapy resources and to improve the patients experience to promote self-management of their hypermobility.

Funding was applied for via the Transformation service. Meeting with the hospital videographer enabled the AHP team to devise a template for the video including education on what is Hypermobility, on the role of exercise - targeted versus general, the benefits of exercise, supportive footwear, pain management, pacing, sleep hygiene, and hand function advice to manage hypermobility. The class exercise programme including hand therapy was videoed using a model.

The video was filmed in half a day. It was filmed using an unscripted question- and- answer format. The Paediatric Rheumatology MDT were filmed in the video. The digital platform is currently with our videography team for further edit. Further signposting to established resources will be included in the Vimeo resource.

Following a final edit, this resource will be used for this patient population group. Feedback from CYP and parents, along with other health professionals is planned.

**Discussion/Results:**

On receipt of the first edit, multi-source feedback from parents/carers, CYP and other health professionals is planned via Microsoft Teams. It is hoped to show the video via Teams and seek anonymized feedback on the Teams chat function. This feedback will be discussed by the Paediatric Rheumatology MDT and will inform the final edit of the Hypermobility video resource.

Further data from Vimeo analytics will be gathered e.g. how many times video watched, how long video is watched for, and what devices are being used.

It is hoped that the Hypermobility Video will help support CYP and families in the long-term management and pain resulting from this condition.

**Key learning points/Conclusion:**

It is hoped that the use of new technologies and innovative ways of delivering care can enable health professionals to implement clinical support, improve self-management of chronic pain related to hypermobility, whilst improving equitable patient access to therapy across the North-West.

Multi-source feedback will help inform the final edit of the video resource. Ongoing evaluation and analysis of Vimeo data will inform whether the resource is a useful adjunct in long term management of pain arising from Hypermobility.

